# Letter regarding “efficacy of esketamine in reducing nausea and vomiting after anesthesia: a systematic review and meta-analysis of randomized controlled trials”

**DOI:** 10.1080/07853890.2026.2624193

**Published:** 2026-02-09

**Authors:** Min Mao, Jing Chen

**Affiliations:** Department of Anesthesiology, Daping Hospital, Army Medical University, Chongqing, China

Dear Editor

We read with great interest the study conducted by Tang et al. [1], which investigated the efficacy of perioperative esketamine in reducing postoperative nausea and vomiting after anesthesia. Based on 38 randomized controlled trials involving 3,425 patients, the authors demonstrated that, compared with controls, patients receiving esketamine experienced a significantly lower incidence of nausea and vomiting, an accelerated time to first flatus, and reduced requirements for rescue analgesia within the first 48 h post-surgery. We commend the authors for their efforts in addressing this important clinical issue. Nevertheless, certain aspects of the study warrant further clarification to strengthen its conclusions.

The inclusion criteria for this study defined the intervention as the perioperative administration of esketamine, with the comparator consisting of standard anesthetic care or placebo (encompassing normal saline or conventional narcotic agents). However, upon detailed examination of the included studies, it was observed that the investigations by Liu et al. [2] and Almenrader et al. [3] employed varying doses of esketamine in the control arm, while the study by Feng et al. [4] inappropriately designated butorphanol in cohort 2 as an experimental intervention. Clearly, these studies or cohorts did not conform to the predefined inclusion criteria and were therefore excluded from the meta-analysis.

In meta-analyses where multiple experimental groups are compared against a single shared control group, repeated inclusion of the control group data violates the assumption of patient data independence, thereby inflating the effective sample size of the control arm and introducing bias into the final pooled result [5]. When assessing nausea and vomiting, the primary outcomes of this meta-analysis, we observed that several studies involved duplicate counting of the control group. Given the similarity of the intervention therapies across these studies, it is methodologically appropriate to combine the intervention groups and perform a single comparison with the shared control group.

To assess the efficacy of esketamine in reducing postoperative nausea and vomiting, we recalculated risk ratios (RRs) along with 95% confidence intervals (CIs) and 95% prediction intervals (PIs) using raw data extracted from the meta-analysis. Studies that did not meet the inclusion criteria were excluded, and data from trials with multiple intervention groups were combined. All analyses were conducted using R software (version 4.3.2; R Core Team). As shown in Figure 1, 20 studies assessed nausea and 22 studies assessed vomiting. The meta-analyses revealed that esketamine was associated with a significant reduction in the risk of nausea (RR = 0.69; 95% CI: 0.50–0.94; *p* = 0.02; *I*^2^ = 75%) and a marginally significant reduction in vomiting (RR = 0.72; 95% CI: 0.51–1.00; *p* = 0.05; *I*^2^ = 71%). However, the prediction intervals spanned from 0.19 to 2.48 for nausea and from 0.18 to 2.81 for vomiting. Since both intervals include the null value of 1, the results suggest considerable uncertainty in the pooled estimates and highlight the need for careful interpretation.

**Figure 1.**
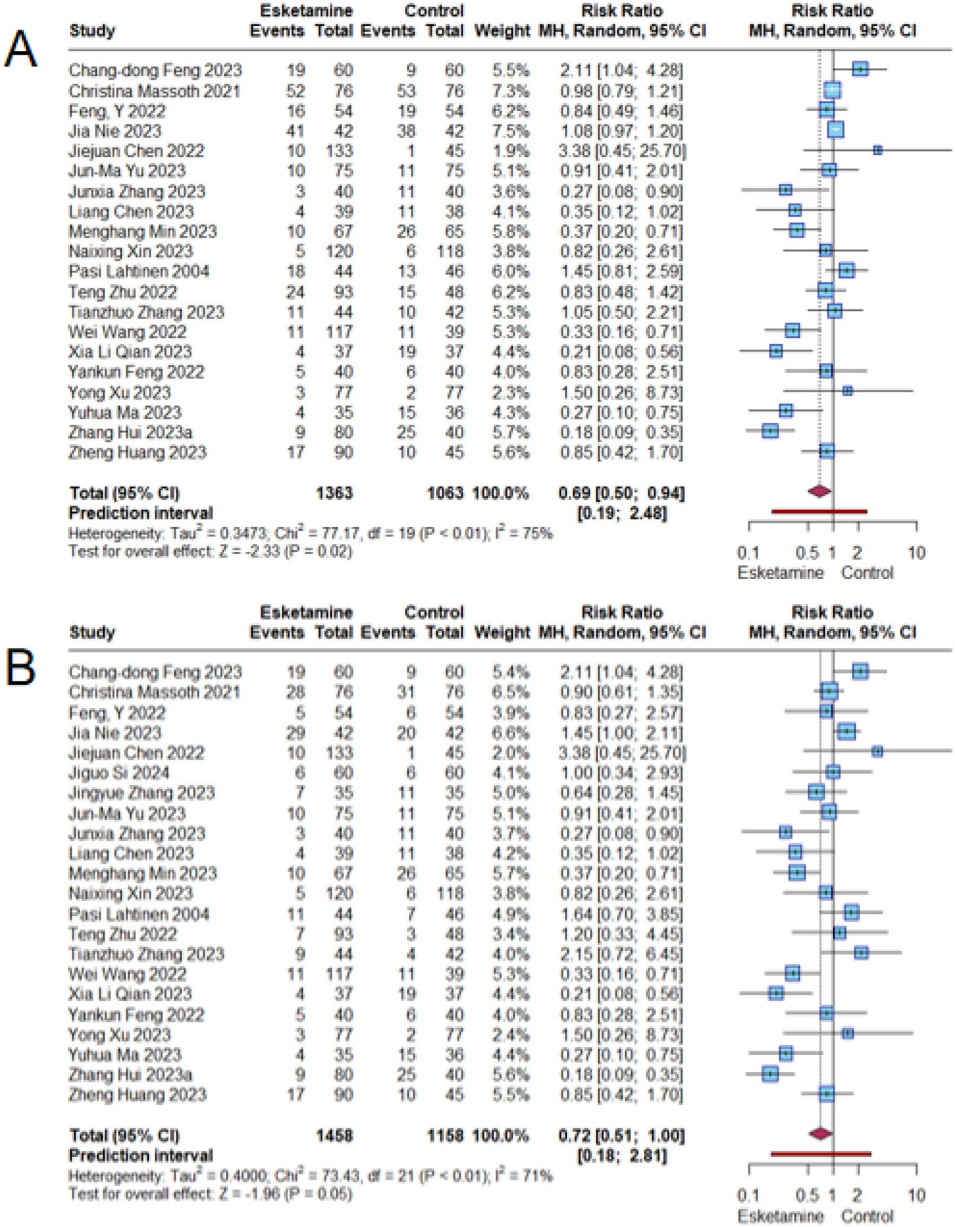
Forest plots showing the effect of esketamine group and control group on postoperative (A) nausea and (B) vomiting.

## Data Availability

All data generated or analyzed during this study are included in this published article.
